# Coding-Complete Genome Sequences of 40 SARS-CoV-2 Omicron XBB, XBB.1, and XBB.2 Sublineage Strains in Bangladesh

**DOI:** 10.1128/mra.00001-23

**Published:** 2023-02-13

**Authors:** Mohammad Tanbir Habib, Mokibul Hassan Afrad, Saikt Rahman, Manjur Hossain Khan, Mohammad Moajjam Hossain, Farhana Khanam, Nicholas R. Thomson, Tahmina Shirin, Firdausi Qadri

**Affiliations:** a Institute for Developing Science and Health Initiatives, Dhaka, Bangladesh; b International Centre for Diarrhoeal Disease Research, Bangladesh, Dhaka, Bangladesh; c Institute of Epidemiology, Disease Control, and Research, Dhaka, Bangladesh; d Department of Biochemistry and Molecular Biology, University of Dhaka, Dhaka, Bangladesh; e Wellcome Sanger Institute, Wellcome Genome Campus, Hinxton, Cambridgeshire, United Kingdom; f London School of Hygiene and Tropical Medicine, London, United Kingdom; Queens College Department of Biology

## Abstract

Here, we report the coding-complete genome sequences of 40 severe acute respiratory syndrome coronavirus 2 (SARS-CoV-2) strains of the newly emerged recombinant Omicron variants XBB, XBB.1, and XBB.2. The strains were isolated from nasopharyngeal swab samples that had been collected from symptomatic patients in Bangladesh between September and October 2022 and were sequenced using an Oxford Nanopore Technologies (ONT) system.

## ANNOUNCEMENT

The severe acute respiratory syndrome coronavirus 2 (SARS-CoV-2) (genus *Betacoronavirus*, family *Coronaviridae*) variant known as the Omicron variant (B.1.1.529) was initially reported to the World Health Organization (WHO) on 24 November 2021 (1, 2). The Omicron sublineage XBB and its descendant XBB.1 (clade 22F) are recombinants of the BA.2.10.1 and BA.2.75 sublineages (3). The XBB.1 sublineage of Omicron was first identified in Singapore in September 2022. Emerging evidence suggests that the XBB.1 sublineage is more immune evading than many other Omicron strains and exhibits resistance to therapeutic monoclonal antibodies (4–7).

As part of a nationwide coronavirus disease 2019 (COVID-19) surveillance program (Institute of Epidemiology, Disease Control, and Research [IEDCR] protocol IEDCR/IRB/2020/11), nasopharyngeal swab specimens were obtained from routine diagnostic sampling from patients across Bangladesh. Viral RNA was extracted from the specimens using the QIAamp viral RNA minikit (Qiagen, Germany). Real-time reverse transcription-PCR (rRT-PCR) was performed using the 2019 novel coronavirus (2019-nCoV) nucleic acid diagnostic kit (Sansure Biotech, China). A total of 40 samples (with threshold cycle [*C_T_*] values of <27) were selected for whole-genome sequencing using the nCoV-2019 sequencing protocol developed by the ARTIC Network (8). ARTIC v3/v4 primer-based multiplex PCR amplicons were generated with Q5 high-fidelity DNA polymerase (New England Biolabs, USA) and used for the sequencing libraries. Amplicons were dA-tailed using the NEBNext Ultra II end repair/dA-tailing module (New England Biolabs, USA) and ligated to native barcodes (EXP-NBD104 and EXP-NBD114) with blunt/TA DNA ligase (New England Biolabs). The resulting sequencing libraries were pooled and sequenced with a FLO-MIN106D flow cell (R9.4.1) for 6 h. Base calling and demultiplexing of raw reads were achieved via MinKNOW v21.02.1. To obtain high-quality genomes, hybrid Illumina/Oxford Nanopore Technologies sequencing is recommended; however, the sequenced reads were assembled utilizing the ARTIC gupplyplex code script with Medaka v1.4 using the EPI2ME desktop agent v3.5.7 (FastQ quality control plus ARTIC plus NextClade) (https://artic.network/ncov-2019/ncov2019-bioinformatics-sop.html). To summarize, 2,685,141 reads were generated (range, 26,251 to 169,436 reads per sample; average length, 491.55 bp for primer panel v4 and 509.06 bp for primer panel v3; read depth, 400 to 2,800×). A nearly complete genome was obtained for each sample, and the genomic information is provided in Table 1. SARS-CoV-2 lineages were assigned according to the proposed nomenclature of Pangolin lineage assignment software v4.1.3 (https://github.com/cov-lineages/pangolin). All tools were run with default parameters.

**TABLE 1:** Data for Bangladesh SARS-CoV-2 Omicron strains

Sample	Date of sample collection (day/mo/yr)	Age (yr)	Sex	Vaccination status	Vaccination type	Booster dose completed	Pangolin lineage	GISAID accession no.	GenBank accession no.	SRA accession no.	No. of reads	Read depth (×)	GC content (%)
icddrb-TND-01-1019	29/9/2022	20	Male	Yes	Sinopharm	No	XBB.1	EPI_ISL_15503005	OQ075381	SRR22799548	103,060	1,714.58	39.7
icddrb-TND-04-2339	29/9/2022	48	Male	Yes	Oxford/AstraZeneca	Yes (Oxford/AstraZeneca)	XBB.1	EPI_ISL_15503006	OQ075382	SRR22799547	136,209	2,267.81	40.2
icddrb-TND-09-0863	29/9/2022	23	Female	Yes	Pfizer-BioNTech	No	XBB.1	EPI_ISL_15503007	OQ075383	SRR22799536	56,065	919.32	41
icddrb-TND-01-1025	1/10/2022	23	Male	Yes	Pfizer-BioNTech	No	XBB.1	EPI_ISL_15503008	OQ075384	SRR22799525	60,456	982.33	40.5
icddrb-TND-04-2353	1/10/2022	46	Male	Yes	Moderna	Yes (Oxford/AstraZeneca)	XBB.1	EPI_ISL_15503009	OQ075385	SRR22799514	90,840	1,505.16	40
icddrb-TND-06-1078	1/10/2022	38	Female	Yes	Oxford/AstraZeneca	Yes (Moderna)	XBB.1	EPI_ISL_15503010	OQ075386	SRR22799513	169,436	2,831.78	40
icddrb-TND-06-1082	1/10/2022	59	Female	Yes	Sinopharm	No	XBB	EPI_ISL_15503011	OQ075387	SRR22799512	83,816	1,377.16	40.1
icddrb-TND-06-1083	1/10/2022	37	Female	Yes	Moderna	No	XBB.1	EPI_ISL_15503012	OQ075388	SRR22799511	88,043	1,456.04	40
icddrb-TND-06-1084	1/10/2022	58	Female	Yes	Sinopharm	No	XBB.1	EPI_ISL_15503013	OQ075389	SRR22799510	64,152	1,056.84	40.4
icddrb-TND-02-0913	2/10/2022	29	Male	Yes	Oxford/AstraZeneca	Yes (Oxford/AstraZeneca)	XBB.1	EPI_ISL_15503014	OQ075390	SRR22799509	36,836	602.17	40.3
icddrb-TND-04-2354	2/10/2022	57	Female	Yes	Oxford/AstraZeneca	Yes (Moderna)	XBB.1	EPI_ISL_15503015	OQ075391	SRR22799546	35,682	564.35	41.8
icddrb-TND-05-1087	2/10/2022	32	Male	Yes	Oxford/AstraZeneca	No	XBB.1	EPI_ISL_15503016	OQ075392	SRR22799545	59,943	989.88	40.3
icddrb-TND-01-1029	3/10/2022	31	Male	Yes	Moderna	Yes (Moderna)	XBB.1	EPI_ISL_15503017	OQ075393	SRR22799544	96,846	1,593.93	40.5
icddrb-TND-05-1094	4/10/2022	25	Female	No	Not vaccinated	No	XBB.1	EPI_ISL_15503018	OQ075394	SRR22799543	61,940	1,004.99	41.2
icddrb-TND-09-0872	6/10/2022	24	Female	Yes	Sinopharm	No	XBB.1	EPI_ISL_15503019	OQ075395	SRR22799542	50,001	818.38	41
icddrb-TND-05-1102	6/10/2022	64	Male	Yes	Sinopharm	No	XBB.1	EPI_ISL_15503020	OQ075396	SRR22799541	61,598	1,003.02	41.5
icddrb-TND-05-1103	6/10/2022	39	Female	No	Not vaccinated	No	XBB.1	EPI_ISL_15503021	OQ075397	SRR22799540	59,370	970.84	40.7
icddrb-TND-04-2380	8/10/2022	27	Female	Yes	Sinopharm	Yes (Oxford/AstraZeneca)	XBB.1	EPI_ISL_15503022	OQ075398	SRR22799539	55,600	913.31	40.8
icddrb-TND-06-1102	12/10/2022	55	Male	Yes	Sinopharm	No	XBB.1	EPI_ISL_15503023	OQ075399	SRR22799538	118,285	1,959.52	40.3
icddrb-TND-04-2414	13/10/2022	19	Male	Yes	Oxford/AstraZeneca	No	XBB.1	EPI_ISL_15503024	OQ075400	SRR22799537	77,933	1,275.05	41.2
icddrb-TND-01-1057	15/10/2022	25	Male	Yes	Pfizer-BioNTech	No	XBB.1	EPI_ISL_15503025	OQ075401	SRR22799535	60,548	999.83	40.7
icddrb-TND-04-2417	15/10/2022	33	Female	Yes	Sinopharm	Yes (Moderna)	XBB.1	EPI_ISL_15503026	OQ075402	SRR22799534	104,377	1,727.02	40.1
icddrb-TND-06-1114	15/10/2022	53	Male	Yes	Oxford/AstraZeneca	Yes (Oxford/AstraZeneca)	XBB.1	EPI_ISL_15503027	OQ075403	SRR22799533	84,263	1,384.36	40.7
icddrb-TND-06-1116	15/10/2022	56	Male	No	Not vaccinated	No	XBB.1	EPI_ISL_15503028	OQ075404	SRR22799532	14,784	2,461.59	39.9
icddrb_TND_09_0850	20/9/2022	36	Male	Yes	Oxford/AstraZeneca	Yes (Pfizer-BioNTech)	XBB	EPI_ISL_15801455	OQ075405	SRR22799531	68,865	1,191.66	39.9
icddrb_TND_04_2295	21/9/2022	60	Male	Yes	Oxford/AstraZeneca	Yes (Moderna)	XBB.1	EPI_ISL_15801456	OQ075406	SRR22799530	81,819	1,407.91	40.3
icddrb_TND_04_2305	24/9/2022	61	Female	Yes	Sinopharm	No	XBB.1	EPI_ISL_15801458	OQ075407	SRR22799529	56,055	956.26	40.3
icddrb_TND_02_0878	25/9/2022	24	Male	Yes	Moderna	Yes (Pfizer-BioNTech)	XBB.1	EPI_ISL_15801459	OQ075408	SRR22799528	78,726	1,353.51	40.3
icddrb_TND_04_2311	25/9/2022	19	Female	Yes	Moderna	No	XBB.1	EPI_ISL_15801460	OQ075409	SRR22799527	38,863	650.80	40.8
icddrb_TND_04_2315	25/9/2022	21	Male	Yes	Sinopharm	No	XBB.1	EPI_ISL_15801461	OQ075410	SRR22799526	34,316	574.48	40.4
icddrb_TND_05_1075	26/9/2022	57	Female	Yes	Sinopharm	Yes (Pfizer-BioNTech)	XBB.1	EPI_ISL_15801462	OQ075411	SRR22799524	50,705	861.80	40.5
icddrb_TND_02_0896	28/9/2022	43	Female	Yes	Moderna	No	XBB.1	EPI_ISL_15801463	OQ075412	SRR22799523	69,544	1,188.77	40.2
icddrb_TND_04_2335	28/9/2022	37	Male	Yes	Sinopharm	No	XBB.1	EPI_ISL_15801464	OQ075413	SRR22799522	55,943	955.42	40.4
icddrb_TND_04_2309	24/9/2022	55	Male	Yes	Oxford/AstraZeneca	No	XBB.1	EPI_ISL_15801467	OQ075414	SRR22799521	49,338	835.16	41.3
icddrb_TND_04_2310	24/9/2022	48	Male	Yes	Sinopharm	No	XBB.1	EPI_ISL_15801468	OQ075415	SRR22799520	26,251	439.75	41.9
icddrb_TND_06_1053	25/9/2022	62	Female	Yes	Oxford/AstraZeneca	Yes (Pfizer-BioNTech)	XBB.2	EPI_ISL_15801469	OQ075416	SRR22799519	63,218	1,082.22	40.7
icddrb_TND_01_1012	27/9/2022	33	Male	Yes	Moderna	Yes (Moderna)	XBB.1	EPI_ISL_15801470	OQ075417	SRR22799518	53,647	914.02	40.6
icddrb_TND_02_0893	28/9/2022	19	Male	Yes	Oxford/AstraZeneca	No	XBB.1	EPI_ISL_15801472	OQ075418	SRR22799517	49,524	842.66	40.7
icddrb_TND_05_1077	28/9/2022	31	Male	Yes	Oxford/AstraZeneca	No	XBB.1	EPI_ISL_15801473	OQ075419	SRR22799516	37,600	636.51	41.1
icddrb_TND_05_1082	28/9/2022	36	Female	Yes	Oxford/AstraZeneca	Yes (Pfizer-BioNTech)	XBB.1	EPI_ISL_15801474	OQ075420	SRR22799515	40,644	698.21	40.2

Relative to the reference genome (Wuhan Hu-1 [GenBank accession number NC_045512.2]), the spike protein-based signature amino acid variations identified 2 of the sequences as sublineage XBB, 37 of the sequences as sublineage XBB.1, and 1 sequence as sublineage XBB.2. The emergence of XBB and its descendants is the result of recombination between BA.2.10.1 and BA.2.75 subvariants of the Omicron variant (9). Therefore, rapid sequencing and sharing of the data can contribute to implementation of quick and informed public health decisions to mitigate COVID-19 consequences.

### Data availability.

The data from this study can be accessed through the GISAID accession numbers listed in Table 1. The NCBI GenBank accession numbers and SRA accession numbers can also be found in Table 1.
